# Short-Term Outcomes of Percutaneous Trephination with a Platelet Rich Plasma Intrameniscal Injection for the Repair of Degenerative Meniscal Lesions. A Prospective, Randomized, Double-Blind, Parallel-Group, Placebo-Controlled Study

**DOI:** 10.3390/ijms20040856

**Published:** 2019-02-16

**Authors:** Rafal Kaminski, Marta Maksymowicz-Wleklik, Krzysztof Kulinski, Katarzyna Kozar-Kaminska, Agnieszka Dabrowska-Thing, Stanislaw Pomianowski

**Affiliations:** 1 Department of Musculoskeletal Trauma Surgery and Orthopaedics, Centre of Postgraduate Medical Education, Professor A. Gruca Teaching Hospital, Konarskiego 13, 05-400 Otwock, Poland; maksymowicz.mm@gmail.com (M.M.-W.); k.kulinski@o2.pl (K.K.); spom@spskgruca.pl (S.P.); 2 Department of Medical Biology, The Stefan Cardinal Wyszynski Institute of Cardiology, ul. Alpejska 42, 04-628 Warsaw, Poland; k.kozar@ikard.pl; 3 Departament of Radiology, Centre of Postgraduate Medical Education in Warsaw, ul. Konarskiego 13, 05-400 Otwock, Poland; dabrowska@poczta.fm

**Keywords:** meniscus, meniscus repair, meniscus tear, trephination, platelet-rich plasma, PRP, chronic meniscal lesion, horizontal meniscal tear

## Abstract

Meniscal tears are the most common orthopaedic injuries, with chronic lesions comprising up to 56% of cases. In these situations, no benefit with surgical treatment is observed. Thus, the purpose of this study was to investigate the effectiveness and safety of percutaneous intrameniscal platelet rich plasma (PRP) application to complement repair of a chronic meniscal lesion. This single centre, prospective, randomized, double-blind, placebo-controlled study included 72 patients. All subjects underwent meniscal trephination with or without concomitant PRP injection. Meniscal non-union observed in magnetic resonance arthrography or arthroscopy were considered as failures. Patient related outcome measures (PROMs) were assessed. The failure rate was significantly higher in the control group than in the PRP augmented group (70% vs. 48%, *P* = 0.04). Kaplan-Meyer analysis for arthroscopy-free survival showed significant reduction in the number of performed arthroscopies in the PRP augmented group. A notably higher percentage of patients treated with PRP achieved minimal clinically significant difference in visual analogue scale (VAS) and Knee injury and Osteoarthritis Outcome Score (KOOS) symptom scores. Our trial indicates that percutaneous meniscal trephination augmented with PRP results in a significant improvement in the rate of chronic meniscal tear healing and this procedure decreases the necessity for arthroscopy in the future (8% vs. 28%, *P* = 0.032).

## 1. Introduction

The menisci are known to play a pivotal role during normal functioning of the knee joint. Their unique and complex chondral structure, as well as their biology, make treatment and repair very challenging. The menisci increase joint stability, distribute load, absorb shock and provide lubrication and nutrition to the remaining joint elements.

Meniscal tears are considered the most common orthopaedic diagnoses. For many years, arthroscopy was regarded as a “gold standard” in therapy with almost 4 million arthroscopies for meniscus pathologies performed annually all over the world, thus representing a serious socio-economic concern with relevant Health Care System costs [1]. Interestingly, more than 50% of these surgeries are conducted in patients older than 45 years with degenerative meniscal lesions [2]. This type of injury is a slowly progressing phenomenon, typically involving horizontal cleavage of the meniscal body with prevalence in the population reaching up to 56%. Interestingly, 61% of those tears have no clinical symptoms of meniscal pathology (pain, aching, stiffness or oedema) [2]. These data provided the background for studies analysing the efficacy of arthroscopy in chronic meniscal lesion therapy. Several randomized clinical trials were performed and demonstrated no additional benefit of partial meniscectomy to sham surgery [3]. These data introduced doubt into the current practice and resulted in making clinical decisions more challenging. Additionally, meniscectomy or partial meniscectomy results in rapid deterioration of articular cartilage and the development of arthritis [4]. Despite the trend of meniscus tear repair and maintaining as much vital tissue as possible [5] there is an inability amongst surgeons to restore anatomical and functional roles of the repaired meniscus. Simultaneously, osteoarthritis progressively develops. These rationales shifted the treatment protocols of chronic meniscal tears into the non-operative manner and motivated the search for new therapeutic strategies.

There are several clinical trials that have provided evidence for the use of blood or bone marrow derived products in the surgical treatment of meniscal pathology: the fibrin clot technique [6,7], platelet-rich plasma (PRP) [8,9] or the bone marrow venting procedure [10,11]. There is, however, no data in the literature evaluating the effect of blood derived products on healing of chronic meniscal tears. Thus we designed a prospective, randomized, double-blind, parallel-group, placebo-controlled study to investigate the effectiveness and safety of minimally invasive (percutaneous) intrameniscal PRP application to complement repair of a symptomatic chronic meniscal lesion. We hypothesized that intrameniscal injection of PRP with concomitant meniscal trephination would result in both an improved healing rate and better functional outcomes.

## 2. Results

**Figure 1:**
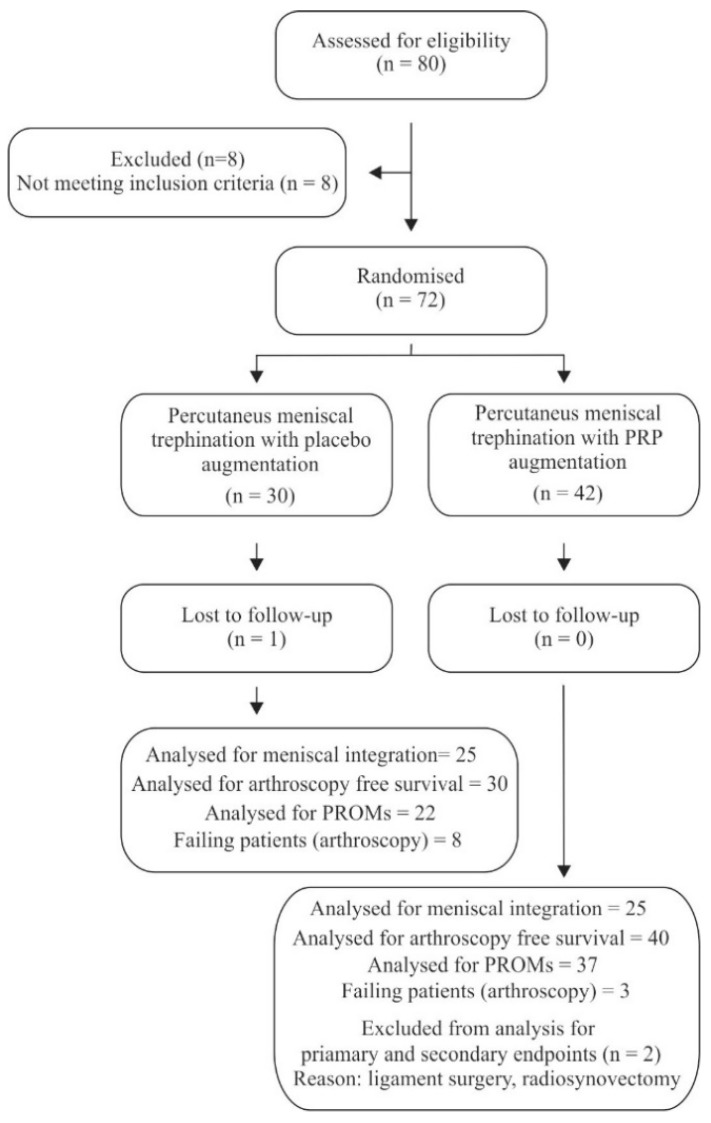
Flow diagram of the trial.

Follow-up ended on 15 January 2019. The median follow-up lasted for 92 weeks (54–157 weeks). 1 patient was lost to follow-up and 2 additional patients were excluded from analysis due to additional procedures (ligament surgery and radio synovectomy) (Figure 1). All remaining patients were functionally assessed at 3, 6, 12 months after the initial procedure. Patients undergoing arthroscopy due to unacceptable quality of life were excluded from analysis of the PROMs. There were no significant differences in baseline characteristics between the groups (Table 1).

### 2.1. Primary Outcome

**Figure 2:**
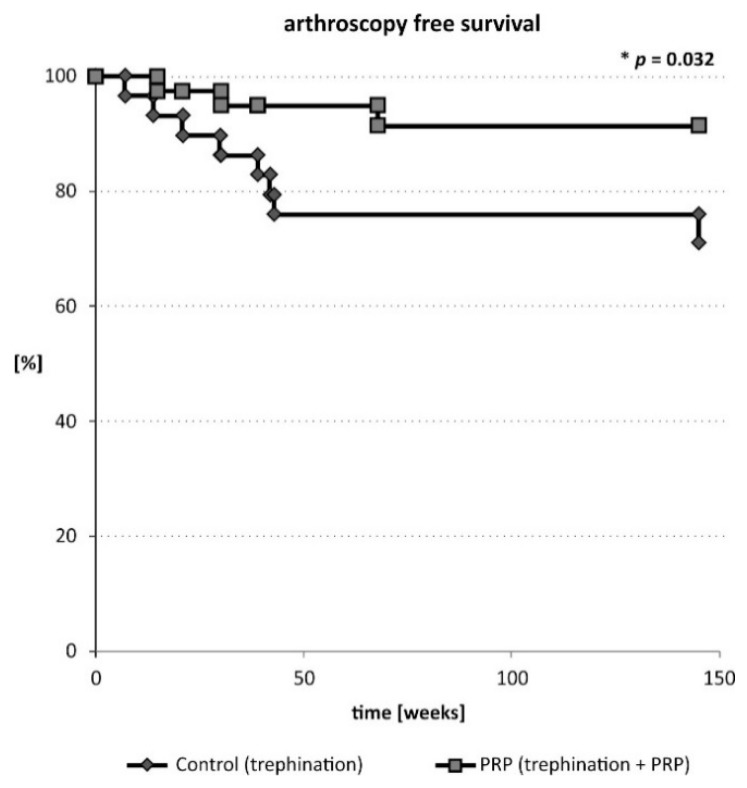
Arthroscopy free survival of patients undergoing trephination of the meniscus with or without PRP augmentation.

Assessment of meniscal healing on MR arthrography was performed at week 33 (13–78) in both groups (Table 2). Induction of the healing process within the meniscus was observed. The healing rate of the meniscal tear, although not significant, was superior in the PRP augmented percutaneous trephination repair group (11 fully and 4 partially healed menisci out of 25 assessed, 60%) than in the control group (7 fully and 4 partially healed menisci out of 26 assessed). When considering cumulative failure rate (arthroscopy and arthrography MRI), the success ratio was significantly better in PRP augmented percutaneous trephination group (*P* = 0.04) (Table 2). In case of 10 patients (8 in the control group and 2 in the PRP augmented group) subsequent arthroscopic meniscectomy or meniscal repair was performed due to unacceptable clinical symptoms. The survival of the PRP injected meniscus (arthroscopy free survival) was superior versus the control group (*P* = 0.032, Figure 2). No significant influence of the number of injected platelets or fold increase in the number of platelets in PRP on meniscal healing was detected.

### 2.2. Secondary Outcomes-Pain

Baseline pain characteristics (VAS and KOOS-pain) of the patients did not differ significantly between groups (Table 3). All patients presented an improvement in pain scores. The changes in VAS and KOOS-pain exceeded minimal clinically important difference (MCID) value in majority of patients (Table 4). We detected a significant difference level in the percentage of patients who benefited by at least MCID in VAS score (39% vs. 65%, *P* = 0.046). No other significant changes were detected.

### 2.3. Secondary Outcomes-Function

Functional outcomes were measured with the IKDC subjective scale, WOMAC and the KOOS subscales (symptoms, function in daily living [ADL], sport/recreation and knee related quality of life [QOL]). Each parameter improved over time in both groups, exceeding the MCID values in vast majority of patients. A significant difference in the percentage of patients who benefited by at least the MCID value in the KOOS Symptoms subscale was detected (48% vs. 76%, *P* = 0.028). We noted that the remaining KOOS subscales, IKDC score and WOMAC score were improved in both groups (Table 3 and Table 4).

### 2.4. Complications

No peri- or post- procedure complications were noted among patients who participated in the final follow-up.

## 3. Discussion

Meniscal healing has always been a major challenge for orthopaedic surgeons. All types of meniscectomies can lead to an increase in the risk of osteoarthritis [15] and evidence comparing the results of total and partial meniscectomy provide data on the beneficial effects of meniscus preservation [16]. The rising problem in meniscal injury treatment is the substantial number of chronic meniscal lesions. Recent studies comparing non-operative and arthroscopic treatment showed no benefit of surgical treatment in large cohorts of patients [3,17]. Data provided by the European Society of Sports Traumatology, Knee Surgery and Arthroscopy [18] or the guidelines published in the British Medical Journal [19] showed no or poor clinical benefit of arthroscopy in the case of degenerative meniscal lesions. In fact, arthroscopy was titled “the last resort” of treatment and applicable due to failure of conservative management.

The most significant finding of this study was that percutaneous trephination with or without a PRP boost induced the healing response of chronic meniscus tears. The process was augmented in the PRP – treated group. Interestingly, our results also demonstrated that no full meniscal integrity is necessary to obtain a clinically important difference in respect to PROMs. Additionally, we found that the functional outcomes (KOOS Symptoms) and pain levels (VAS) scored higher in patients treated with PRP-augmentation than in the control group.

For this study we used leukocyte- and platelet-rich plasma (L-PRP). Its fluid like state enables delivery to the target site by needle injection. Once activated, L-PRP forms a gel and releases most of the growth factors in the first few hours post injection until fully dissolved within 3 days [20]. It supports growth factors to act as an assembly of platelets and leukocytes in a complex matrix. Although leukocyte and platelet rich fibrin (L-PRF), was shown to slowly release growth factors over a period of about 7 days [21] providing optimal kinetics of a release, it forms a 3D matrix that cannot be delivered via a minimally invasive way (e.g., intra-articular injection)

PRP has been shown to influence not only the process of meniscal healing in vitro and in vivo [22,23] but also the treatment of other musculoskeletal injuries [24,25]. Some evidence has been provided for the use of PRP in meniscal repair [8,9]. The authors found that clinical outcomes and healing rates were better with the introduction of PRP into the lesion at the end of surgery. Griffin et al. performed a retrospective chart review with a minimum of 2 year follow-up and failed to show any benefit of PRP augmentation [26]. However, the study was underpowered for the primary and secondary outcomes. Another Study by Strümper, R. et al. demonstrated that intra-articular autologous conditioned serum injection might be an effective treatment option for knee pain associated with meniscal lesions [27]. The authors showed that surgery was avoided during the 6-month observation period and the Oxford Knee Score improved significantly from 29.1–44.3 in 83% of patients. Interestingly, the structural findings on MRI, measured by Boston Leeds Osteoarthritis Knee Score, also showed significant improvement. The limitations of the study were its retrospective character and lack of control group analysis. We believe that an additional weak point of this study was connected to not addressing perimeniscal capillary plexus (PCP) while performing the joint injection. Trephination is a known technique usually employed during arthroscopy [28,29]. It involves the formation of vascular access channels from the meniscus periphery (PCP) to the tear. This process initiates bleeding into the meniscal lesion and subsequent tissue repair response. This simple technique was showed to increase the meniscal healing rate while applied during a surgical procedure [30], most probably by providing the injury site with both growth factors and mesenchymal stem cells.

The results of experimental studies support the hypothesis that PRP may improve meniscal healing through activation of fibrochondrocytes present within the meniscus [31]. The process also involves the activity of mesenchymal stem cells, which seem to be necessary for the repair of meniscal lesions [23]. The PRP itself releases the “cytokine cocktail” of the healing cascade [25]. The main growth factors are: platelet derived growth factor, platelet derived endothelial growth factor, vascular endothelial growth factor, insulin like growth factor, platelet derived angiogenesis factor, transforming growth factor-b, hepatocyte growth factor and others [32]. This release initiates the chemotaxis of immunocompetent cells, inflammation, angiogenesis and as a consequence the process of synthesis of the extracellular matrix and tissue remodelling. The PRP works at various levels for joint homeostasis. Studies have shown that PRP application decreases catabolism while increasing anabolic activity and observations have been made that catabolic activity in meniscus chondral tissue helps identify patients who are at risk for progression of osteoarthritis [33]. Other processes, such as chondral remodelling is promoted by PRP administration. Higher production of collagen II, matrix molecules and prostaglandin has been observed in hyaline cartilage [34,35]. On the contrary, Lee et al. showed on a rabbit model of a circular meniscal defect that PRP treatment failed to enhance the production of meniscus cartilage. Additionally, it accelerated fibrosis and increased catabolic processes [36]. However, findings from in vivo and in vitro studies cannot be directly translated to clinical practice.

Increasing data provide evidence for the necessity of mesenchymal stem cells in delivering the positive effect of PRP on healing of meniscal and hyaline cartilage defects [23,37] and the process of chondrocyte differentiation [38]. PRP has been shown to enhance proliferation of stromal stem cells [39] as well as their adhesion and migration [40]. This phenomenon is probably dependent on the release of a growth factor cocktail and triggering of synovial tissue to create a more balanced intra-articular environment. Recent studies link the synovium-derived stem cells to chondral regeneration, as they possess chondrogenic potential and encouraging results have been shown for cartilage repair purposes in experimental studies [41]. 

We hypothesize, that trephination, by creating multiple wounds and inducing intrameniscal bleeding, starts the process of tissue repair with activation of synovial and blood derived stem cells, which—in our study—are stimulated by addition of PRP. The combination of those two processes allows for efficient meniscal tissue regeneration.

### 3.1. Strengths

This is the first study to employ percutaneous trephination of a chronic meniscal lesion with or without PRP augmentation. The second strength is the study design itself, the randomized and blinded nature of this study and being adequately powered to detect differences in healing rates. Lastly, independent evaluators were used for assessing of the outcomes.

### 3.2. Limitations

We acknowledge some limitations in this study. The study group was small, increasing the risk of type II error. Additionally, some patients refused MRI arthrography due to its interventional character, still their comfort of life improved significantly. Also, calculation of the primary outcome might have been influenced by factors that could affect MRI images and their interpretation. There is also the issue of heterogeneity within groups. Localization of the tear in medial or lateral compartments may influence the primary outcome, as the biology of those menisci might differ. We find no statistically significant differences between these groups but in the literature the results are mixed [42]. Additionally, PROMs data have partially overlapping 95% confidence intervals, increasing the risk of type II error. Moreover, it is still unknown which of the factors are solely responsible for the improved outcomes in the PRP group. The rehabilitation protocol was uniform in all patients but we could not control those differences that might have occurred in patients being treated in multiple outpatient centres. Lastly, the observation period in this study allowed only for a short-term analysis.

## 4. Materials and Methods

### 4.1. Trial Design and Informed Consent

This was a parallel-group, superiority trial with equal randomization. The study protocol was approved by an appropriate Institutional Review Board and was publicly accessible before enrolment of the first patient. We performed the study in accordance with the ethical standards outlined in the 2013 revision of 1975 Declaration of Helsinki and we report the results according to the 2010 CONSORT statement. The potential benefits and risks of meniscal trephination, PRP injection and follow-up were explained to each study patient. All patients provided written informed consent for participation in this study and no patient declined to participate. Clinical Trial Registration: The study protocol was approved by Bioethics Committee at Centre of Postgraduate Medical Education (36/PB/2013 approved on 29.05.2013) and was publicly accessible before enrolment of the first participant. The clinical trial databases at cmkp.edu.pl-36/PB/2013, clinicaltrials.gov-NCT03066583.

### 4.2. Eligibility Criteria

Patients were recruited from a single public knee clinic at a tertiary care, university health centre between 2016 and 2018 (Figure 1). 72 patients with chronic (horizontal) meniscal lesions were enrolled: 30 were randomized to undergo percutaneous trephination (control group) and 42 were randomized to undergo percutaneous trephination with PRP injection at the repair site. Detailed inclusion and exclusion criteria are presented in Table 5.

### 4.3. PRP and Thrombin Preparation

PRP and its activator (thrombin) was prepared as in Reference [9]. In this study we used Red-l-PRPIIB-1 according to the new classification system [43]. PRP was prepared by a dedicated laboratory assistant in the BL2 facility. Briefly, the PRP preparation procedure involved drawing of 120 mL of venous blood and centrifuging the blood using a refrigerated centrifuge in a two-step process. First, the PRP layer was isolated, including a “buffy coat” and a small fraction of underlying red blood cells (900 rpm × 9 min). Additional centrifugation and isolation of PRP was then applied (3200 rpm × 15 min). The preparation was packed into sterile vials labelled with the patient ID. In the study group, 6–8 mL of PRP solution was used, while in the control group, 6–8 mL of sterile 0.9% saline was applied. Right before application, PRP was activated using 20 mM CaCl_2_ (Teva, Basel, Israel) and 25 IU/mL autologous thrombin. It was then injected into the tear site of the meniscus with a double chamber syringe. Platelets and leukocyte concentration were assessed for each sample.

### 4.4. Procedures

All procedures were performed by the same senior orthopaedic surgeon under ultrasound guidance (R.K.) in the outpatient department. In brief, the PRP or control solution was prepared as described above. Local anaesthetic was used. After identification of a horizontal tear via ultrasound, the needle was introduced into the tear lesion (passing through the PCP, red zone, red-white zone and white zone) with continuous injection of studied solutions (starting while in the PCP). 5–10 separate needle introductions through all layers were performed. After discharge, patients were referred to outpatient physiotherapy units and encouraged to follow a unified rehabilitation protocol. In short, all patients wore a hinged knee brace for 4 weeks. Exercises with a range of motion from 0 to 90 degrees for 6 weeks were encouraged. Weight bearing as tolerated was allowed - beginning from day 1. Early quadricep muscle activation was initiated. At 6 weeks post procedure, a low-resistance stationary bicycle and one-quarter body weight leg presses were initiated. Additional increases in low-impact knee exercises were permitted as tolerated starting at 12 weeks post procedure.

### 4.5. Outcomes

The primary outcome was meniscus healing assessed using 1.5T magnetic resonance imaging (MRI) arthrography with a dedicated knee coil (Siemens, Erlangen, Germany). Meniscus healing was evaluated by two independent attending radiology consultants, who were blinded to the patient allocation. We did not notice any intra-observer bias. Complete healing was considered when full meniscus integrity was noted during MR arthrography (no intrameniscal contrast media). Partial healing was considered with contrast media filling a defect between 1–3 mm. Healing failure was considered when contrast media was detected within the meniscal body. Additionally, failure was defined as performing arthroscopic meniscectomy or meniscal repair. Arthroscopy free survival was analysed.

Secondary outcomes (patient reported outcome measures–PROMS) included pain assessment with the visual analogue scale (VAS) and functional outcome assessment with the Knee injury and Osteoarthritis Outcome Score (KOOS), Western Ontario and McMaster Universities Osteoarthritis Index (WOMAC) and International Knee Documentation Committee Subjective Knee Evaluation (IKDC) [12,44,45]. All secondary outcomes were assessed before the procedure and at 3, 6, 12, 24 months post injection. Minimally clinical important difference (MCID) was assessed for PROMs [13,14,46,47]. Patients were closely monitored for complications. There were no changes to the protocol during study duration.

### 4.6. Randomization

The randomization list for allocating patients to the study groups was generated using the “simple randomization” function on the StatSoft GraphPad QuickCalcs web site (http://www.graphpad.com/quickcalcs) [48]. We used sequentially numbered, opaque, sealed envelopes to conceal the allocation. Patients were consecutively enrolled and assigned to the study groups. Intervention assignment was performed during PRP preparation.

### 4.7. Blinding

The patients, the data collectors and the assessors were blinded to the intervention type.

### 4.8. Statistical Analysis

We used the R statistical package (www.rproject.org) for statistical analyses [49]. Differences in meniscus healing rates were assessed through analysis of a contingency table using Fisher’s exact test. All categorical data were analysed using Fisher’s exact test. The VAS score, KOOS, WOMAC and IKDC score were analysed using the two-tailed Mann-Whitney *U* test or unpaired *t*-test (after assessment for parametric or non-parametric distribution using the Shapiro-Wilk test) [50]. Arthroscopy-free survival was analysed using Kaplan Meyer plot and log-rank testing for statistical significance. Results were considered statistically significant at a *P*-value < 0.05. Sample size was calculated for the primary outcome (meniscus healing), with a two-tailed significance level at alpha = 0.05 and beta = 0.8, assuming a difference in the meniscus healing rate of 15% between the study groups according to the method described in Reference [51] and based on previous studies [52,53]. Minimum recruitment level was estimated to be 28 patients per group. Assuming an attrition or non-compliance rate of 10% during the study, we aimed to recruit at least 30 patients per group.

## 5. Conclusions

Our blinded, prospective, randomized, controlled trial on the role of PRP and percutaneous trephination of the chronically torn meniscal tissue indicates that percutaneous trephination of the meniscal tissue is an effective technique improving meniscal integrity as well as PROMs. The augmentation of this technique with PRP results in a significant improvement in the rate of meniscal healing (52% vs. 30%, *P* = 0.04). Importantly, this simple procedure seems to decrease the necessity for arthroscopy in the future (8% vs. 28%, *P* = 0.032). This study showed that PRP augmentation could provide significant and clinically important benefits. Further studies in this field are encouraged. The risk of adverse events related to percutaneous trephination with augmentation with PRP is very low.

## Figures and Tables

**Table 1:** Baseline characteristics of study patients in the control and PRP-treated groups.

	Control Group (*n* = 30)	PRP-Treated Group (*n* = 42)	*P*-Value
Age (years)	46 (27–68)	44 (18–67)	*P* = 0.31
Sex (M:F)	19:11	22:20	*P* = 0.25
BMI (range)	28 (21–36)	27 (19–37)	*P* = 0.27
Kellgren-Lawrence scale (0 grade:1 grade:2 grade)	23:7:0	30:12:0	*P =* 0.79
PRP (PLT × 10^3^/μL)	732 (220–1586)	823 (320–1659)	*P =* 0.16
Meniscus (MM:ML)	30:0	41:1	*P* = 0.58

Data are presented as median (range) or mean ± standard error (confidence interval (CI) 95%) unless otherwise indicated. BMI, body mass index; PRP, platelet rich plasma; PLT, platelets; MM—medial meniscus; ML—lateral meniscus.

**Table 2:** Primary outcome assessment.

Cumulative Outcome (Assessed Using MRI and Arthroscopy) (*P* = 0.04)		
Outcome	PRP-treated group (*n* of menisci)	Control group (*n* of menisci)
Healed	10	5
Partially healed	4	3
Failed	13	19
MRI (*P* = 0.41)		
Outcome	PRP-treated group (*n* of menisci)	Control group (*n* of menisci)
Healed	11	7
Partially healed	4	4
Failed	10	15

MRI, magnetic resonance imaging; PRP, platelet-rich plasma.

**Table 3:** Patient-reported outcome measures (pain: VAS and KOOS-pain; function: IKDC, WOMAC, KOOS: symptom, ADL, sport/recreation and QOL).

	Control Group		PRP Group		
PROM	Pre-Procedure	Post Trephination	Pre-Procedure	Post Trephination	*P* ^a^
VAS	4.40 ± 0.07 (3.55–5.25)	2.05 ± 0.08 (1.27–2.82)	5.38 ± 0.05 (4.77–5.99)	1.97 ± 0.05 (1.40–2.55)	0.39
IKDC	54.92 ± 0.54 (49.08–60.77)	88.12 ± 0.89 (79.97–96.28)	51.99 ± 0.34 (47.62–56.36)	85.98 ± 0.52 (79.79–92.16)	0.36
WOMAC	28.93 ± 0.61 (22.42–35.45)	7.50 ± 0.59 (2.06–12.94)	34.36 ± 0.35 (29.90–38.82)	9.72 ± 0.32 (5.95–13.48)	0.21
KOOS					
Pain	65.30 ± 0.54 (59.51–71.10)	89.00 ± 0.63 (83.19–94.81)	57.48 ± 0.30 (57.18–57.78)	87.24 ± 0.36 (82.99–91.48)	0.22
Symptoms	69.86 ± 0.62 (63.18–76.54)	90.42 ± 0.56 (85.26–95.58)	63.53 ± 0.39 (63.23–63.83)	92.03 ± 0.27 (88.80–95.26)	0.27
ADL	68.42 ± 0.66 (61.33–75.50)	92.38 ± 0.61 (86.80–97.95)	63.70 ± 0.37 (63.40–64.00)	89.36 ± 0.36 (85.07–93.64)	0.25
S/R	33.50 ± 0.62 (26.84–40.16)	78.98 ± 1.10 (68.83–89.12)	35.83 ± 0.51 (35.53–36.14)	69.52 ± 0.77 (60.29–78.74)	0.11
QoL	35.00 ± 0.49 (29.73–40.27)	68.18 ± 1.08 (58.28–78.08)	37.90 ± 0.26 (37.59–38.20)	67.06 – 0.55 (60.56–73.56)	0.42

^a^ For the control group vs. PRP group; Data are presented as mean ± standard error (CI 95%) unless otherwise indicated. PROM, patient related outcome measures; VAS, visual analogue scale; WOMAC, Western Ontario and McMaster Universities Osteoarthritis Index; IKDC, International Knee Documentation Committee; KOOS, Knee injury and Osteoarthritis Outcome Score; ADL, activities of daily living; S/R, sport/recreation; QOL, quality of life.

**Table 4:** Patient-reported outcome measures (pain: VAS and KOOS-pain; function: IKDC, WOMAC, KOOS: symptom, ADL, sport/recreation and QOL).

		Control Group		PRP Group			
PROM	MCID	Mean Change	Improved by at Least MCID [%]	Mean Change	Improved by at Least MCID [%]	*P* ^a^	*P* ^b^
VAS	2 [12]	2.36 ± 0.0.09 (3.86–5.20)	39	3.62 ± 0.07 (2.82–4.43)	65	0.027	0.046
IKDC	16.7 [13]	33.66 ± 0.84 (25.95–41.36)	83	34.74 ± 0.55 (28.17–41.31)	78	0.48	0.48
WOMAC	11.5 [14]	21.77 ± 0.67 (15.65–27.90)	65	24.77 ± 0.37 (20.40–29.14)	86	0.16	0.053
KOOS							
Pain	16.7 [13]	24.95 ± 0.62 (19.24–30.66)	65	29.50 ± 0.45 (24.18–34.81)	73	0.17	0.36
Symptoms	17.4 [13]	18.38 ± 0.82 (10.81–25.95)	48	27.93 ± 0.42 (22.89–32.96)	76	0.016	0.028
ADL	18.4 [13]	24.61 ± 0.74 (17.79–31.43)	57	26.27 ± 0.39 (21.67–30.87)	76	0.18	0.1
S/R	12.5 [13]	43.75 ± 1.12 (33.43–54.07)	83	34.65 ± 0.76 (25.57–43.74)	70	0.12	0.22
QoL	15.6 [13]	32.67 ± 1.06 (22.93–42.41)	70	28.43 ± 0.52 (22.23–34.64)	76	0.29	0.41

^a^ For mean changes; ^b^ for % of patients improved by at least MCID. Data are presented as mean ± standard error (CI 95%) unless otherwise indicated. PROM, patient related outcome measures; VAS, visual analogue scale; WOMAC, Western Ontario and McMaster Universities Osteoarthritis Index; IKDC, International Knee Documentation Committee; KOOS, Knee injury and Osteoarthritis Outcome Score; ADL, activities of daily living; S/R, sport/recreation; QOL, quality of life; MCID, Minimal Clinically Important Difference.

**Table 5:** Inclusion and exclusion criteria.

Inclusion Criteria	Exclusion Criteria
skeletally mature patients aged 18–70 years chronic horizontal tears on MRI tear located in the vascular/avascular portion of the meniscus single tear of the medial and/or lateral meniscus	arthritic changes (Kellgren-Lawrence scale >2) discoid meniscus axial leg deformity (valgus > 6 deg)- concomitant chondral defects (> 2 ICRS) Inflammatory diseases (rheumatoid arthritis) chondral defects above ICRS 2 on MRI

MRI, magnetic resonance imaging.
